# Emergency Department “Bounce-Back” Rates as a Function of Emergency Medicine Training Year

**DOI:** 10.7759/cureus.10503

**Published:** 2020-09-17

**Authors:** Janine Curcio, Andrew Little, Chelsea Bolyard, Anand Gupta, Michelle Secic, Meenal Sharkey

**Affiliations:** 1 Emergency Medicine, OhioHealth Doctors Hospital, Columbus, USA; 2 Research, OhioHealth Research Institute, Columbus, USA; 3 Biostatistics, OhioHealth Research Institute, Columbus, USA

**Keywords:** bounce-backs, residency training, emergency medicine training

## Abstract

Introduction: Since the 1990s, the emergency department (ED) unscheduled return visit (URV), or “bounce-back,” has been used as a quality of care measurement. During that time, resident training was also scrutinized and uncovered a need for closer resident supervision, especially of second-year residents. Over the years, bounce-backs have continued to be analyzed with vigor, but research on residency training and supervision has lagged with few studies concurrently investigating residency supervision and bounce-backs. Other literature on resident supervision suggests that with adequate attending supervision, resident performance is equivalent to attending performance. With that in mind, it was hypothesized that resident bounce-back rates will be equivalent to attending bounce-back rates, and there will be no change among residency years. The primary objective of this study was to determine the rate at which patients are seen as a bounce-back visit within 72 hours of their initial visit to a community hospital ED during the study time frame. The secondary aims were to evaluate if the ED bounce-back rate is impacted by training level (residents or attending) and to describe bounce-back patient characteristics, including primary complaint/disease, age, comorbidities and issues with compliance.

Methods: A retrospective chart review of 1000 charts was conducted from September 2015 to September 2017. Charts were randomly selected by the Quality & Patient Safety (QPS) team and, after applying inclusion/exclusion criteria, 732 charts were analysed. Inclusion criteria included age ≥ 18 years, patients treated by an Emergency Medicine (EM) resident during their initial visit and patients with a “discharge” disposition. Exclusion criteria included patients seen as a scheduled return visit (e.g., two-day return for blood pregnancy recheck, wound check, etc.). Demographics, initial visit variables, comorbidities and bounce-back data were collected based on electronic record query or chart review. Data was analysed using means, standard deviations, medians and ranges for continuous variables. Logistic regression modelling techniques were used to examine factors that affect whether the patient had a bounce-back visit.

Results: The rate of URVs within 72 hours of the patient's initial visit was 4.65%. PGY1 and PGY2's bounce-back rate was 3.8% and 3.6%, respectively, and PGY3 and PGY4's bounce-back rate was 5.7% and 5.6%, respectively (p-value=.63). There was no statistically significant change among residency years. Most bounce-back characteristics analysed including primary complaint, age, and comorbidities demonstrated no statistical significance in the increased rate of bounce-back except for patients with a history of tobacco abuse, alcohol abuse and chronic pain. Current smokers were 6.5 times more likely to bounce back than former smokers (odds ratio=6.485, 95% confidence interval = 2.089 to 20.133, p-value=0.0012) and those with chronic pain were 2.5 times more likely to bounce back than those without chronic pain (odds ratio=2.518, 95% confidence interval =1.029 to 6.164, p=0.0431).

Conclusion: EM residency training year does not increase the frequency of bounce-backs in a community hospital ED. Finally, patients with substance abuse and chronic pain were more likely to bounce back.

## Introduction

In Emergency Medicine (EM), a patient who returns for an unscheduled visit to the emergency department (ED) shortly after initial discharge (e.g., within 2-30 days) is called a “bounce-back” or an unscheduled return visit (URV). Since 1990, multiple studies reported on the association between ED bounce-backs and ED or patient metrics, including quality of care, patient insurance status, patient age, ED overcrowding and patient satisfaction [1-6]. Despite nearly 30 years of reporting on this subject, there is still no unified definition of the bounce-back timeframe; reports range from 24 hours to “undefined” [7]. In the classical paper, “Bounces,” Pierce et al. reported that 3% of patients seen at the ED returned within two days of primary visit; these short-term return visits could be split into four categories: patient-related, physician-related, disease-related, and system-related [8].

The rate of ED bounce-back is often used as a quality of care indicator. Studies indicate that only 5%-20% of return visits are related to inadequate medical care at the primary visit [9]. Other factors that contribute to bounce-backs include advanced age [10], high-grade triage/illness severity [9,10] and number of comorbidities [11]. Bounce-back patients are reported to have an increased rate of adverse events, including inpatient admission or death [9,12].

However, a paucity of data exists on the impact of junior or resident physician training on bounce-back rates and patient outcomes [13]. In the EDs of community teaching hospitals, EM residents may perform the majority of care under the supervision of attending physicians. Sacchetti et al. determined that 4% of patients seen by second-year residents needed “major” modifications to their treatment plans, and 33% required “minor” modifications [14]. They concluded that close attending supervision is required for second-year residents, including direct examination of the patient. Supporting this, van der Leeuw et al. reviewed the effects of residency training on patient outcomes and determined that residents have similar patient outcomes compared to faculty when there is dedicated supervision [15]. As such, bounce-back rates should be similar in patients treated by residents at any level, as all should be directly supervised by an attending physician.

The purpose of this study is to add to the body of knowledge regarding patient bounce-back in the ED, especially related to patients who are treated by resident physicians. Furthermore, we are interested in bounce-back rates of patients seen by residents as a metric to evaluate the level of attending supervision in a community teaching hospital.

## Materials and methods

Study design

We conducted a retrospective chart review during a 24-month study period after approval of our Institutional Review Board.

Study setting and population

This study was performed in a midwestern community hospital with 75,000 annual patient visits. This hospital houses a four-year EM residency. The study population included eligible patients who presented during a 24-month study period (September 2015 through September 2017). Inclusion criteria included age ≥ 18 years, patients treated by an EM resident physician during their initial visit, and patients with a discharge disposition following the initial ED visit. Exclusion criteria included patients seen at a follow-up visit as a scheduled return (e.g., two-day return for antibiotics, two-day return for blood pregnancy recheck, wound check, 24-hour abdominal re-examination, etc.), patients treated by clinicians other than the EM resident during first visit (e.g., attending physicians, nurse practitioners, etc.), patients whose initial visit disposition was admission to hospital or transfer to another facility and patients who left the ED prior to final disposition or against medical advice (AMA).

Study protocol

We reviewed a randomly selected subgroup of 1000 patients. The charts were selected by the hospital system's Quality and Patient Safety (QPS) team based on inclusion/exclusion criteria. ED bounce-backs within 72 hours were recorded and may have occurred at any system ED with any type of practitioner. We collected the demographics, initial visit variables, comorbidities and ED provider data. All data was uploaded and stored in the Research Electronic Data Capture (REDcap) database [16,17]. After charts were queried and unblinded, trained reviewers reviewed 10% of charts for query accuracy. The bounce-back charts were then reviewed by the principal investigator to enter the “bounce-back” variables. These characteristics were entered after both the index visit’s chart and bounce-back visit’s chart were reviewed [9]. If a new chief complaint was listed and previous complaint not discussed in the History of Present Illness, the bounce-back was deemed unrelated. If the medical decision-making statement specifically discussed returning for worsening or persistent symptoms in 24 to 48 hours, it was determined that the return was associated with following return precautions.

Measures

Our primary aim was to calculate the ED bounce-back rates of patients treated at the initial visit by resident physicians and evaluate if ED bounce-back rate is impacted by training level. Our secondary aim was to describe bounce-back patient characteristics, including primary complaint/disease, age, comorbidities, issues with compliance, etc.

Data analysis

As our primary hypothesis is that the residents have an equal rate of bounce-back, we were not able to conduct a sample size calculation for this study. Instead, we chose 1000 charts as we subjectively estimated would be an adequate sampling. The ED bounce-back rates of patients treated by resident physicians were calculated based on the variable ED bounce-back criteria. Based on these results, the bounce-back rates were broken down separately for clinical knowledge, by resident level and attending experience level.

Demographics, initial visit variables, comorbidities, and ED provider data were described for the two groups (bounce-back yes/no) using means, standard deviations, medians, and ranges for continuous variables. The discrete variables were described using frequencies and percentages. Logistic regression modeling techniques (bivariate and multivariate) were used to examine which factors affect whether the patient has a bounce-back visit.

## Results

**Figure 1:**
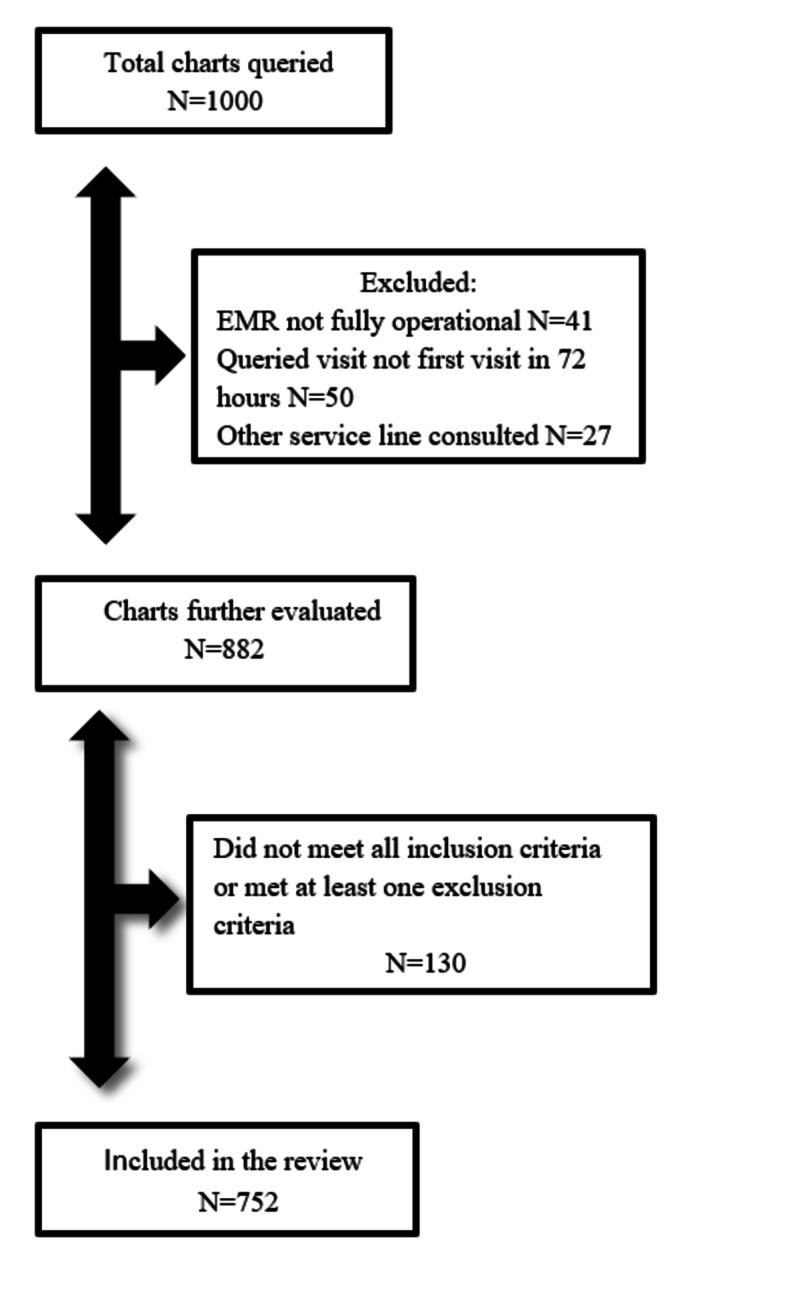
Inclusion and exclusion criteria EMR, electronic medical record

We initially queried 1000 charts. After charts were queried, it was determined to limit the charts to FY16 and FY17 as our electronic medical record system did not fully become operational until August of 2015. Our ability to fully query all variables would be limited on any charts dated before then. Using our exclusion and inclusion criteria, a total of 130 patients were excluded and 752 patients met the criteria for inclusion in this study (Figure 1). Of these 752 patients, 35 bounced back within 72 hours, for an overall bounce-back rate of 35/752 = 4.65%.

The breakdown of the bounce-back rates by resident and attending physicians and level/experience is shown in Table 1. For residents, the bounce-back rate was about 4% for PGY1 and PGY2 and then approximately 5.5% for PGY3 and PGY4. For attendings, the bounce-back rate was about 6% for less than 24 months, 3% between two and five years, and 5% after five years. However, these results were not statistically significant.

**Table 1:** Bounce-back rates by resident and attending experience

Physician	Level/experience	Did not bounce back	Bounced back	p-value
Resident	PGY1	152/158 (96.2%)	6/158 (3.8%)	0.63
	PGY2	216/224 (96.4%)	8/224 (3.6%)	
	PGY3	164/174 (94.3%)	10/174 (5.7%)	
	PGY4	185/196 (94.4%)	11/196 (5.6%)	
Attending	<24 months	61/65 (93.9%)	4/65 (6.1%)	0.50
	>2 to <5 years	134/138 (97.1%)	4/138 (2.9%)	
	>5 years	522/549 (95.1%)	27/549 (4.9%)	

When analysing the bounce-back qualities (Table 2), most bounce-backs occurred within 48 hours of the index visit. Of our 35 bounce backs, 77% were related to their index visit chief complaint and 37% returned following return precautions given by the index visit provider. Only four (11%) required hospital admission.

**Table 2:** Assessment of bounce-back variables – bivariate logistic regression ED, emergency department

Characteristic	Statistic/category	Did not bounce back (n=717)	Bounced back (n=35)	p-value
Days between initial visit and bounce back	0	--	3/35 (8.6%)	--
	1	--	12/35 (34.3%)	--
	2	--	18/35 (51.4%)	--
	3	--	2/35 (5.7%)	--
Chief complaint				
Chest pain	Checked	0 (0.0%)	2/35 (5.7%)	--
Abdominal pain	Checked	0 (0.0%)	7/35 (20.0%)	--
Neurological concern (e.g., headache)	Checked	0 (0.0%)	4/35 (11.4%)	--
Pulmonary concern (e.g., cough)	Checked	0 (0.0%)	5/35 (14.3%)	--
Other	Checked	0 (0.0%)	26/35 (74.3%)	--
Return related to initial visit	Yes	--	27/35 (77.1%)	--
Returned to ED following return precautions	Yes	--	13/35 (37.1%)	--
Return to ED due to adverse event related to initial treatment	No	--	35/35 (100.0%)	--
ED disposition following bounce-back	Admission to hospital	--	4/35 (11.4%)	--
	Discharge	--	27/35 (77.1%)	--
	Other	--	4/35 (11.4%)	--

Next, we explored what factors impact bounce-back rates using bivariate logistic regression, as summarized in Table 3. Smoking history, alcohol abuse and chronic pain were the only factors found to be statistically significantly related to bounce-back in that analysis. Furthermore, after a multivariate logistic regression analysis using a model with the factors statistically significant along with the resident and attending experience variables, only smoking and chronic pain were related to bounce-back rates as listed in Table 4.

**Table 3:** Assessment of factors and their relationship with bounce-back rates – bivariate logistic regression ICD, International Classification of Diseases; IVDA, intravenous drug abuse; MI, myocardial infarction

Characteristic	Statistic/category	Did not bounce back (n=717)	Bounced back (n=35)	p-value
90 years or older?	Yes	1/717 (0.1%)	0 (0.0%)	--
Gender	Female	475/717 (66.2%)	24/35 (68.6%)	--
	Male	242/717 (33.8%)	11/35 (31.4%)	0.78
Insurance status	Insured	616/717 (85.9%)	32/35 (91.4%)	--
	Uninsured	101/717 (14.1%)	3/35 (8.6%)	0.36
Quarter	Q1	151/717 (21.1%)	10/35 (28.6%)	--
	Q2	187/717 (26.1%)	13/35 (37.1%)	0.18
	Q3	181/717 (25.2%)	4/35 (11.4%)	--
	Q4	198/717 (27.6%)	8/35 (22.9%)	--
Length of stay	N	717	35	--
	Mean ± SD	11117.9 ± 4981.06	11626.3 ± 6356.55	0.56
	Range	1680.0-33480.0	2520.0-32040.0	--
	Median	10440	10740	--
Chief complaint				
Chest pain		54/717 (7.5%)	3/35 (8.6%)	0.82
Abdominal pain		165/717 (23.0%)	6/35 (17.1%)	0.42
Neurological concern (e.g., headache)		78/717 (10.9%)	5/35 (14.3%)	0.53
Pulmonary concern (e.g., cough)		53/717 (7.4%)	5/35 (14.3%)	0.14
Other		451/717 (62.9%)	22/35 (62.9%)	0.99
Medical history				
Diabetes	Yes	105/717 (14.6%)	4/35 (11.4%)	0.60
Hypertension	Yes	200/717 (27.9%)	13/35 (37.1%)	0.24
Hyperlipidemia/hypercholesterolemia	Yes	115/717 (16.0%)	8/35 (22.9%)	0.29
End-stage renal disease as defined by the ICD	Yes	4/717 (0.6%)	1/35 (2.9%)	0.14
Smoking history	Current	152/691 (22.0%)	17/35 (48.6%)	--
	Former	238/691 (34.4%)	4/35 (11.4%)	0.001
	Never	301/691 (43.6%)	14/35 (40.0%)	--
Chronic obstructive pulmonary disorder	Yes	52/717 (7.3%)	5/35 (14.3%)	0.13
Asthma	Yes	131/717 (18.3%)	7/35 (20.0%)	0.80
Chronic pain	Yes	66/717 (9.2%)	8/35 (22.9%)	0.01
Active cancer	Yes	46/717 (6.4%)	2/35 (5.7%)	0.87
Coronary artery disease/history of MI	Yes	32/717 (4.5%)	4/35 (11.4%)	0.07
Heart failure	Yes	21/717 (2.9%)	2/35 (5.7%)	0.36
IVDA	Yes	16/717 (2.2%)	2/35 (5.7%)	0.21
Alcohol abuse	Yes	13/717 (1.8%)	3/35 (8.6%)	0.02
Social work/counseling consult recommended	No	0 (0.0%)	23/23 (100.0%)	--
	Yes	2/2 (100.0%)	0 (0.0%)	--
	Missing	715	12	--

**Table 4:** Assessment of factors and their relationship with bounce-back rates – multivariate logistic regression

Parameter	Comparison	Estimate	Standard error	Test statistic	p-value	Odds ratio	Confidence interval	
							Lower limit	Upper limit
Intercept	Intercept	-2.2299	0.4036	30.5235	--	--	--	--
Smoking	Never vs. current	-0.7019	0.3911	3.2205	0.0727	--	--	--
	Current vs. former	1.8696	0.578	10.4632	0.0012	6.485	2.089	20.133
	Never vs. former	1.1677	0.582	4.0258	0.0448	3.215	1.027	10.057
Chronic pain	Yes vs. no	0.9236	0.4567	4.0898	0.0431	2.518	1.029	6.164
Alcohol	Yes vs. no	0.976	0.7304	1.7855	0.1815	--	--	--

## Discussion

In 1992, Sacchetti et al. concluded that attendings should be directly involved in the patient care of all patients seen by a resident physician, specifically a second-year, in the ED [14]. It was determined that 37% of patient plans needed modifications whether major or minor. Accreditation Council for Graduate Medical Education (ACGME) Common Program Requirements state, “Each patient must have an identifiable and appropriately-credentialed and privileged attending physician (or licensed independent practitioner as specified by the applicable Review Committee) who is responsible and accountable for the patient’s care.” It states that supervision can be direct or indirect depending on the scenario [18]. Over the years, EM programs have accepted the standard that an attending will supervise every resident patient interaction regardless of the severity, but as a resident progresses in training, attending involvement in a patient’s case becomes less and less as the resident becomes more efficient and capable on his or her own. We sought to address the question, “Does training resident physicians affect the bounce-back rate of an institution and does this rate change depending the PGY year?” Up until now, bounce-backs have been analysed to measure quality of care and patient metrics, but never been used to assess residency training and corresponding supervision.

Theoretically, if the standard of EM education is that an attending supervises every resident-patient interaction, we hypothesized that our “bounce-back” rates were similar across residency year and equivalent to the average for an attending physician. Essentially, if a residency year had a statistically significant difference in the amount of bounce-backs, it could suggest a lack of adequate supervision for that group of residents for their level of learning and training. Fortunately, our data supported our hypothesis with our overall bounce-back rate being 4.65% with a statistically insignificant difference between residency classes. It was also similar to our attending bounce-back rate that was between 3% and 6%.

Our study has several limitations. The first is that we had a reduced sample size based on logistics and resource availability. Due to resources within our system, the number of charts that could feasibly be reviewed were significantly reduced. Although 1000 charts suggested a strong sample size for our initial calculations, it did limit our secondary calculations, and they may have been underpowered. Also, the only bounce-backs that could be accounted for were ones that returned to our system. We were unable to follow any bounce-backs that may have returned to another hospital system in the city. Granted, our hospital system includes several hospitals in small proximity to each other making this of low likelihood, but there is a chance that our reported bounce-back rate is lower than the actual bounce-back rate of our community.

Secondly, most of our data was pulled directly from the chart by our QPS team. While this limits bias, we may have inaccuracies based on the quality of medical charting. If the patient’s medical conditions or substance abuse history was not charted in our electronic medical record, it would not have been detected by our QPS team and wouldn’t have been analysed. This does not affect the primary aim of our study, but may further skew our secondary aim of attempting to describe bounce-back patient characteristics. The only information that was unable to be queried by our QPS team was the three variables relating to the characteristics of the bounce-back visit: return related to the initial visit, returned to ED following return precautions and return to ED due to an adverse event related to initial treatment. This was entered by the principal investigator, who was unblinded to the study and therefore could introduce bias.

## Conclusions

We concluded that the rate of ED bounce-backs at a community EM residency site is similar across residency years with no statistically significant change. Based on this, we can infer that attendings are providing adequate supervision at each level of training. Of note, it is also confirmed that patients with substance abuse disorders and chronic pain are more likely to bounce back.
